# Exchangeable Zinc Pool Size Reflects Form of Zinc Supplementation in Young Children and Is Not Associated with Markers of Inflammation

**DOI:** 10.3390/nu14030481

**Published:** 2022-01-22

**Authors:** Julie M. Long, Afsana Mim Khandaker, Rahvia Alam Sthity, Jamie E. Westcott, Andrei Matveev, Robert E. Black, Janet C. King, Kazi Munisul Islam, Shams El Arifeen, Tahmeed Ahmed, M. Munirul Islam, Christine M. McDonald, Nancy F. Krebs

**Affiliations:** 1 Department of Pediatrics, University of Colorado Denver Anschutz Medical Campus, Aurora, CO 80045, USA; julie.long@cuanschutz.edu (J.M.L.); jamie.westcott@cuanschutz.edu (J.E.W.); 2 Nutrition and Clinical Services Division, International Centre for Diarrhoeal Disease Research, Bangladesh (icddr,b), Dhaka 1212, Bangladesh; akhandaker@nevada.unr.edu (A.M.K.); rahviaalam@gmail.com (R.A.S.); munisul@icddrb.org (K.M.I.); shams@icddrb.org (S.E.A.); tahmeed@icddrb.org (T.A.); mislam@icddrb.org (M.M.I.); 3 Department of Mathematical and Statistical Sciences, University of Colorado Denver, Denver, CO 80204, USA; andrei.matveev@ucdenver.edu; 4 Institute for International Programs, Bloomberg School of Public Health, Johns Hopkins University, Baltimore, MD 21218, USA; rblack1@jhu.edu; 5 International Zinc Nutrition Consultative Group, University of California, San Francisco, CA 94158, USA; jking829@berkeley.edu (J.C.K.); christine.mcdonald@ucsf.edu (C.M.M.); 6 Division of Nutritional Sciences and Toxicology, University of California, Berkeley, CA 94720, USA; 7 Division of Gastroenterology, Hepatology and Nutrition, Department of Pediatrics, School of Medicine, University of California, San Francisco, CA 94158, USA

**Keywords:** EZP, zinc isotopes, inflammation, Bangladesh, biomarker, serum zinc

## Abstract

A sensitive and reliable biomarker of zinc status has yet to be identified, but observational research suggests that the exchangeable zinc pool (EZP) size may be a possible biomarker. This randomized, placebo-controlled trial aimed to compare the change in EZP size from baseline to endline in 174 children who were preventatively supplemented with 10 mg of zinc as part of a multiple micronutrient power (MNP) or as a standalone dispersible tablet for 24 weeks versus a placebo powder. The effects of systemic inflammation on EZP size were also evaluated. Zinc stable isotopes were administered intravenously to children at baseline and endline, and the EZP was measured by the urine extrapolation method. A total of 156 children completed the study with the zinc dispersible tablet group having the greatest increase in EZP (14.1 mg) over 24 weeks when compared with the MNP group (6.8 mg) (*p* < 0.01) or placebo group (2.0 mg) (*p* < 0.001). Median EZP size was not different between children with normal or elevated serum inflammatory markers. EZP size was responsive to longitudinal zinc supplementation and reflected the expected difference in bioavailability for two forms of supplementation. The apparent absence of an effect of inflammation on EZP size may offer an advantage for use as a biomarker for group comparisons between different interventions.

## 1. Introduction

A sensitive and reliable biomarker of zinc status remains elusive. Serum or plasma zinc and proxy indicators such as dietary zinc intake and stunting are most commonly used to assess zinc status at the population level, but each have limitations that make evaluation of zinc status challenging [1]. Another putative measure of zinc status is the exchangeable zinc pool (EZP), or the size of the combined pools in the body that readily exchange with zinc in the plasma within 2–3 days [2]; the zinc in liver is a major portion of the EZP in children. This rapidly exchanging zinc has been estimated to include approximately 10% of total body zinc and its availability is proposed to be critical for zinc normal metabolism and homeostasis. The attraction to measuring zinc status via EZP is that previous studies in adults, including controlled depletion and repletion studies, and from a limited number of studies in infants suggest that EZP correlates with dietary zinc intake and with absorbed zinc [2,3]. Additionally, unlike serum or plasma zinc, which is a negative acute phase reactant, EZP may be unaffected by systemic inflammation, making it a potentially more robust marker for zinc status [4]. 

While EZP may have utility as a zinc biomarker, studies to date that reported EZP in children have primarily been small, observational, cross-sectional, and conducted over a wide age range. To determine the extent to which EZP responds to changes in zinc intake and reflects zinc status, large randomized clinical trials are needed [5]. The zinc in powders trial (ZiPT) provided an opportunity to prospectively measure the effect of different forms of zinc supplementation on changes in EZP in a study population of young children at risk for zinc deficiency [6,7]. 

The primary objective of this sub-study was to compare the change in EZP from baseline to endline in children who were preventively supplemented with zinc as part of a multiple micronutrient power (MNP) consumed with food or as a zinc dispersible tablet for 24 weeks versus a placebo powder. We hypothesized that bioavailability of the zinc in a dispersible tablet would be more favorable compared to that in the MNP and would result in the highest EZP at the end of the intervention. The secondary objective was to measure the effect of systemic inflammation on EZP at baseline. 

## 2. Materials and Methods

### 2.1. Study Design

This was a sub-study of the randomized, partially blinded, multi-arm, placebo-controlled zinc in powders trial (ZiPT) [6,7]. The primary aim was to compare the change in the EZP size from baseline to endline in a subgroup of children at risk for zinc deficiency who were preventively supplemented with 10 mg of zinc as part of an MNP added to a single meal each day, as a standalone daily dispersible tablet, vs. a placebo powder for 24 weeks. EZP was measured by the urine extrapolation method as previously described [2]. The study was approved by the UCSF Benioff Children’s Hospital Oakland Institutional Review Board, Colorado Multiple Institutional Review Board, and International Centre for Diarrhoeal Disease Research, Bangladesh (icddr,b) Institutional Review Board, and was registered at clinicaltrials.gov NCT03406793. This research was carried out following the rules of the Declaration of Helsinki.

### 2.2. Participants

A total of 174 children between the ages of 9 to 11 months from the peri-urban community of Mirpur in Dhaka, Bangladesh were enrolled between September 2018 and July 2019. Participants were enrolled if they were 9–11 months old, had a weight-for-length z-score (WLZ) ≥ −3, were enrolled in the primary ZiPT, and were randomized to one of the following groups: daily 10 mg zinc MNP; daily 10 mg dispersible zinc tablet; or daily placebo powder. Detailed description of the randomization process has been previously outlined in the primary outcomes paper of the parent study [6,7]. Exclusion criteria included severe acute malnutrition as defined by a WLZ < −3 and/or the presence of bipedal edema and/or a mid-upper arm circumference of <115 mm, congenital anomalies or any other conditions that interfere with feeding, or chromosomal anomalies and other health problems (e.g., jaundice, tuberculosis, etc.). Written consent was obtained from the participant’s mother or legal guardian prior to enrollment and commencement of study procedures. Fifty-eight participants were assigned to each of three intervention groups detailed below.

### 2.3. Power and Sample Size

The sample size calculation for this sub-study of ZiPT was determined by the sample size of the biochemical subgroup of the main trial (*n* = 58 per group). This number was determined to have 80% power to detect a difference in EZP size of 0.67 mg/kg body weight, assuming a one-sided test, alpha of 0.05, equal standard deviation across groups of 1.2, and potential loss of 15% of participants due to attrition or sampling errors. This effect size is consistent with findings from a study of zinc supplementation in Pakistani infants, which reported a difference of 0.8 mg/kg body weight for EZP size [8]. 

### 2.4. Intervention

Participants were assigned to one of three different interventions at enrollment. The first group received a daily MNP with 10 mg of elemental zinc and 6 mg of iron with vitamins A, C, D, E, B12, thiamine, riboflavin, niacin, folate, pyridoxine, copper, selenium, and iodine (DSM India Private Limited, Haryana, India). The second group received 10 mg elemental zinc in a dispersible tablet (Nutriset, Malaunay, France) given daily. The final group of children were given a daily placebo powder that did not contain any vitamins or minerals. Additional information about the interventions has been previously published [6].

### 2.5. Preparation and Administration of IV Zinc Isotopes

Intravenous zinc stable isotopes of ^67^Zn and ^70^Zn (Trace Science International, Toronto, ON, Canada) were prepared in the Pediatric Nutrition Lab on the University of Colorado Anschutz Medical Campus (CU Anschutz) in Aurora, CO, USA. Isotope solutions were tested and found to be sterile and negative for fungus and pyrogens prior to shipment to Dhaka and administration to participants. Participants fasted (including breast milk) for at least two hours prior to isotope administration. At 8 a.m. on study day 1, ~300 µg of accurately measured zinc stable isotope was administered to children via the antecubital vein using sterile techniques. Researchers collected any isotope losses that occurred during administration on ashless filter paper and analyzed the paper for isotope enrichment and total zinc to accurately adjust the amount of dose administered. These procedures and sample collections were repeated after 24 weeks of the assigned intervention. The order of which isotope, ^67^Zn or ^70^Zn, was given to each participant at baseline and endline was randomized to ensure no bias was introduced by the order the isotope was administered.

### 2.6. Sample Collections

On Study Day 0, participants and their mothers arrived at the clinical trials unit (CTU) on the icddr,b campus. Upon admission to the CTU, a baseline spot urine sample of ~30 mL was collected using procedures to avoid zinc contamination [9]. On Study Days 4 thru 7, field research assistants visited the participant’s household once in the morning and once in the afternoon to collect additional spot urine samples (~30 mL each). The urine samples were stored in −20 °C freezers at icddr,b until shipment to CU Anschutz for sample purification and analyses.

On Study Day 1, immediately before the administration of the IV isotope and through the same antecubital access, a 5 mL blood sample was collected for serum Zn and inflammatory markers. Serum was separated 30 min after the collection, and aliquots stored at −20 °C until shipment and analysis of samples.

### 2.7. Sample Analyses

Urine samples were processed and analyzed in the Pediatric Nutrition Lab for zinc isotope enrichment. The urine samples were digested via a microwave digester system to destroy organic compounds and zinc was subsequently separated from inorganic compounds via chelation and extraction. Zinc isotope ratios were measured via inductively coupled plasma mass spectrometry (ICP-MS, Agilent 7700x, Santa Clara, CA, USA) and converted to enrichment [3]. Serum zinc was also measured in the Pediatric Nutrition Lab via ICP-MS. Serum C-reactive protein (CRP) and alpha-1-acid glycoprotein (AGP) were analyzed via sandwich ELISA in the VitMin Laboratory in Germany [10].

### 2.8. Data Calculations and Statistical Analyses 

Anthropometric z-scores were calculated using the World Health Organization Anthro program, version 3.2.2, (Geneva, Switzerland). To calculate the size of EZP, urine enrichment from the four days of sample collection was natural log transformed and data points were plotted to obtain the linear regression of the log percent enrichment using Graph Pad Prism Version 9 (San Diego, CA, USA). The slope of the robust regression was extrapolated to the y-intercept to determine the size of EZP for a participant at time zero of the zinc isotope infusion [2]. EZP normalized to body weight is also presented in mg of zinc per kg of body weight.

Statistical analyses were completed with GraphPad Prism and R [11]. All data were checked for completeness, normalization, and outliers. Outliers were identified using the z-score method. One-way analysis of variance (ANOVA) was used to compare the change in EZP by group over the intervention period. ANCOVA analysis and Mann–Whitney tests were used to assess the effects of inflammation on EZP status. Analyses were considered statistically significant if a *p*-value of 0.05 or less was observed. All values are presented as mean (SD) unless otherwise noted.

## 3. Results

**Figure 1:**
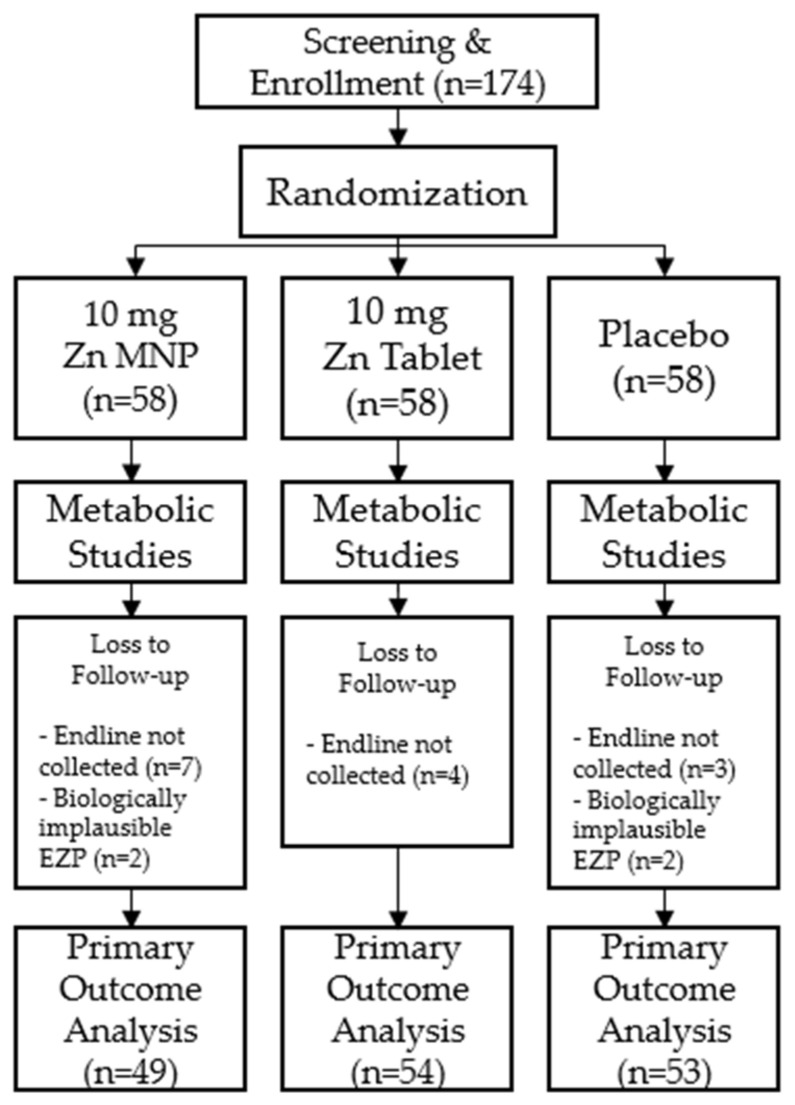
Consort diagram of study participants.

Of the 174 participants enrolled in the study, 156 participants successfully completed both time points of the study and were included in analyses. A total of eighteen participants were excluded from final analyses; 14 did not return for the endline study visit after 24 weeks of supplementation and 4 had biologically implausible EZP measurements (Figure 1). Baseline demographic and anthropometric measurements were not significantly different by group at baseline (Table 1). 

**Figure 2:**
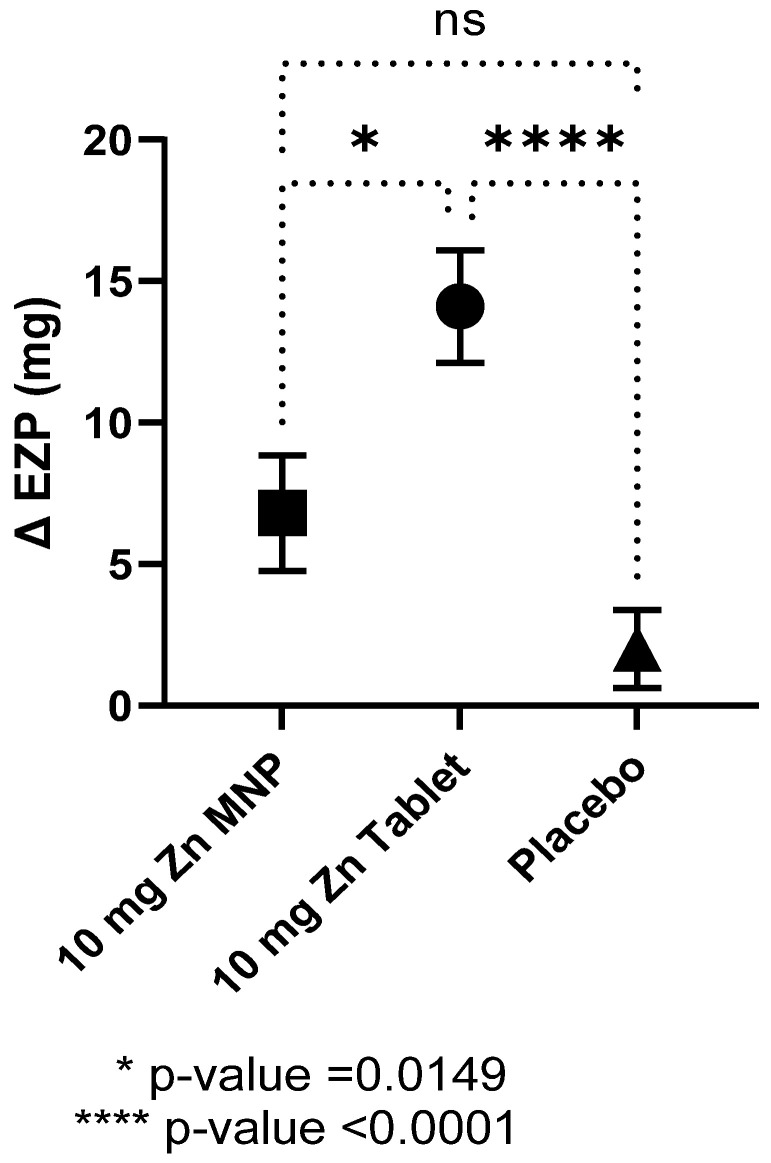
Mean (SEM) change in the size of the exchangeable zinc pool (EZP) by group after 24 weeks of supplementation. Statistical differences between the MNP group and tablet group and between the tablet and placebo group, as determined by ANOVA and Tukey’s multiple comparison tests. MNP = multiple micronutrient powder, Zn = zinc.

Baseline EZP did not differ by group and all individual data points were within the proposed normal range for 9–11 months of age (Table 2) [5]. After 24 weeks of zinc supplementation, the mean EZP in the dispersible tablet group was significantly higher compared to placebo group, while that of the MNP group also increased but did not reach statistical significance (Table 2). In contrast, no differences between the mean baseline and endline EZP were observed in the placebo powder group. One-way ANOVA analysis revealed statistical differences between the three groups (*p* < 0.0001) with post hoc analysis showing statistical differences between the tablet and MNP group and between the tablet and placebo groups (Figure 2). The dispersible tablet had the greatest increase in EZP at endline compared to the other two groups. 

**Figure 3:**
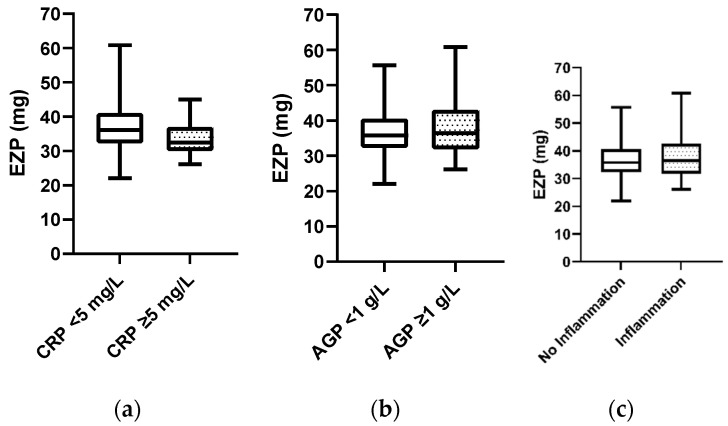
(**a**) Comparison of median exchangeable zinc pool (EZP, mg) at baseline for participants with normal serum C-reactive protein (CRP) status (*n* = 143) vs. participants with elevated CRP (≥5 mg/L) (*n* = 12), determined by Mann–Whitney test; *p*-value = 0.07. (**b**) Comparison of median EZP (mg) at baseline for participants with normal alpha-1-acid glycoprotein (AGP) status (*n* = 122) vs. participants with elevated AGP (≥1 g/L) (*n* = 33), determined by Mann–Whitney test; *p*-value = 0.96. (**c**) Comparison of median EZP (mg) at baseline for participants with normal CRP and AGP status (*n* = 120) vs. participants with either elevated CRP and/or AGP (*n* = 35), determined by Mann–Whitney test; *p*-value = 0.78.

Mann–Whitney analysis revealed no apparent difference in EZP at baseline when serum CRP and/or AGP was elevated compared to when the inflammatory markers were within the normal range (Figure 3). Additionally, analysis using a mixed model regression approach to examine CRP and AGP as continuous variables revealed similar results. A positive correlation between serum zinc and EZP was observed (R^2^ = 0.08, *p* = 0.0001). 

## 4. Discussion

To our knowledge, this was the first study to prospectively measure changes in EZP size after zinc supplementation in a large randomized controlled trial. This research demonstrated that the size of the EZP significantly increased after 24 weeks of zinc supplementation from a dispersible tablet, while an equal amount of zinc provided in MNP and consumed with meals did not result in a significant increase. Our hypothesis was supported, that the group that consumed the zinc in a dispersible tablet mixed with water or breastmilk would have the greatest increase in EZP due to more favorable bioavailability. Serum zinc concentrations demonstrated a pattern of change that was similar to the changes in EZP for the three groups. These findings indicate that, on average, the size of EZP reflected changes in zinc status associated with a period of supplementation. Additionally, EZP size did not significantly differ in children with evidence of systemic inflammation compared to children without inflammation. 

The increase in the EZP of children who were supplemented with zinc as a dispersible tablet in comparison to children who consumed the placebo powder supports previous findings that EZP is responsive to increased intake and total absorbed zinc [2,3]. For the current study, the smaller change in EZP when zinc was consumed in the MNP form is consistent with earlier findings in toddlers from the same community, where environmental enteric dysfunction is highly prevalent, that zinc absorption from MNP mixed with foods is relatively impaired [9,12]. Healthy breastfed infants in the U.S. who were similar in age to those in the current study and who were randomized to different sources of zinc in complementary foods, were found to have no differences in mean EZP or plasma zinc concentrations among feeding groups despite a two-fold difference in daily intake over approximately 3 months. However, EZP (but not plasma zinc) was significantly correlated with both dietary zinc intake and daily absorbed zinc [3]. 

Reference cut-offs for EZP size have not been established. Miller et al. [5] tentatively proposed a normative value of 4 mg/kg and a lower cutoff value of 2 mg/kg for infants and children aged 8 to 120 months. The values were derived from zinc absorption studies in children in which participants had a daily absorbed zinc at or above the estimated physiologic requirement for age proposed by the Institute of Medicine for Dietary Reference Intakes [13]. According to this proposed normative value, the means of EZP size of the study participants were within this range, and none of the participants had EZP size of 2 mg/kg or less. The sample size for this EZP sub-study is insufficient to examine associations with functional outcomes, e.g., incidence of infectious morbidities or growth. 

In this study, we did not detect effects of inflammation on EZP. Group comparisons of children with elevated serum AGP and/or CRP versus children with values within the normal ranges demonstrated no statistical differences in EZP at baseline. The apparent absence of association between inflammation and EZP contrasts with serum zinc. As a negative acute phase reactant, serum zinc is known to be reduced in the presence of inflammation, particularly among young children [14,15]. EZP may not be affected by inflammation in the same way since the liver is a major component of the EZP. The liver sequesters zinc from circulation in the presence of systemic inflammation, but because the liver is included in the EZP, changes in the distribution of zinc throughout the pools would have little to no effect on the overall EZP size [16]. 

Strengths of this study include that the zinc isotope administration and urine collections were completed under closely supervised conditions, which greatly decreased the risk of environmental zinc contamination during sample collections and increased sample integrity. The study population, by design, had a narrow age and weight range, which is important to the current analyses because EZP size is associated with both age and body size [5]. The large sample size and high study retention ensured that study power was met. 

The results of this analysis support the utility of EZP as a potentially useful biomarker of the response of zinc status to an intervention under research conditions. However, its use for assessment of zinc status for routine community surveillance or for an individual’s assessment is likely to be limited. The apparent lack of influence of inflammation on EZP size, especially from chronic, low-grade inflammation reflected by AGP, suggests EZP may be particularly advantageous as a biomarker in settings with a high prevalence of systemic inflammation. A recent comprehensive analysis of observational studies of EZP in children and the factors that influence its size concluded that child weight, age, and weight-for-age were the strongest predictors of EZP size. Normal and low cut-off values of EZP relative to body weight were proposed but the absence of demonstrated diagnostic utility was acknowledged. In contrast, predictors of EZP size in adults additionally included absorbed zinc, sex, and plasma zinc concentration, and experimental data were sufficient to propose a normative model of EZP and a 95% prediction interval [5]. The results of the current study provide new insight regarding plausibility for use of EZP size as a marker of zinc status. More research would be required to determine whether factors in a growing child distinctly alter the distribution of zinc among rapidly versus more slowly exchanging body pools compared to adults. 

EZP determinations require technical training for the administration of isotopes and collection of samples. It is also considerably more time consuming to measure than serum/plasma zinc because the urine extrapolation method requires multiple urine samples versus one blood draw for serum zinc. In addition, specific instrumentation and technical expertise is needed to measure isotope ratios and determine isotopic enrichment. All of these factors contribute to a greater expense and specialized skillset required to measure EZP. 

## 5. Conclusions

In conclusion, these results from a subsample of a large randomized controlled trial of zinc supplementation confirmed that the size of the EZP was increased after 24 weeks of supplementation with a dispersible zinc tablet. Similar to responses in serum zinc among the study groups, participants receiving 10 mg zinc in a dispersible tablet had the greatest change in EZP compared to the same dose of zinc in MNP consumed with food or to the placebo groups. These data suggest that EZP size provides a potentially useful ancillary index of zinc status that may be relatively resistant to changes associated with inflammation. 

## Figures and Tables

**Table 1:** Baseline demographic and anthropometric data of Bangladeshi children by intervention group.

	10 mg Zn MNP (*n* = 49)	10 mg Zn Tablet (*n* = 54)	Placebo (*n* = 53)
Sex, % male	48%	48%	51%
Age, mo	9.4 (1.1)	9.2 (1.1)	9.5 (1.0)
Length, cm	68.7 (2.5)	69.1 (2.8)	68.8 (2.7)
Weight, kg	7.6 (0.9)	8.0 (1.1)	7.7 (1.0)
Length-for-age z-score	−1.5 (1.0)	−1.2 (1.1)	−1.5 (1.1)
Weight-for-age z-score	−1.3 (1.0)	−0.9 (1.1)	−1.2 (1.1)
Weight-for-length z-score	−0.7 (0.9)	−0.3 (1.0)	−0.5 (1.0)

Values presented as mean (SD). ANOVA analysis revealed no statistical differences between groups. MNP = multiple micronutrient powder; Zn = zinc.

**Table 2:** Mean (SD) of the exchangeable zinc pool (EZP), serum zinc, and systemic inflammatory markers at enrollment and after 24 weeks intervention by group.

	10 mg Zn MNP (*n* = 49)	10 mg Zn Tablet (*n* = 54)	Placebo (*n* = 53)	*p*-Value
**Baseline**				
EZP, mg	37.7 (6.2)	38.0 (7.8)	35.6 (7.8)	0.190
EZP, mg/kg	5.0 (0.7)	4.9 (1.1)	4.6 (0.9)	0.074
Serum zinc, µg/dL *	71.5 (10.0)	67.3 (13.2)	69.9 (15.6)	0.266
AGP, g/L *	0.7 (0.4)	0.8 (0.5)	0.7 (0.4)	0.292
CRP, mg/L *	1.4 (3.0)	2.2 (5.4)	0.9 (1.8)	0.205
**Endline**				
EZP, mg	44.5 (13.4) ^a^	52.1 (16.5) ^b^	37.6 (9.1) ^a^	<0.0001
EZP, mg/kg	5.3 (1.6) ^a^	5.9 (1.9) ^b^	4.4 (1.0) ^a^	<0.0001
Serum zinc, µg/dL **	83.1 (16.3) ^a^	95.5 (22.1) ^b^	74.2 (16.2) ^a^	<0.0001
AGP, g/L ***	0.8 (0.4)	0.7 (0.3)	0.8 (0.4)	0.367
CRP, mg/L ***	1.8 (4.1)	2.0 (4.5)	2.4 (4.5)	0.783
Δ EZP, mg	6.8 (14.3) ^a^	14.1 (14.7) ^b^	2.7 (10.6) ^a^	<0.0001
Δ EZP, mg/kg	0.3 (1.8) ^a^	1.1 (1.9) ^b^	−0.2 (1.2) ^a^	0.0004
Δ Serum zinc, µg/dL****	15.1 (27.4) ^a^	28.2 (25.0) ^b^	4.4 (18.3) ^a^	<0.0001

Values presented as mean (SD), all pairwise comparisons between groups statistically significant at *p* < 0.05; means with distinct superscript letters in rows are statistically different *p* < 0.05. * MNP Group: *n* = 49, Tablet Group: *n* = 54, Placebo Group: *n* = 52. ** MNP Group: *n* = 47, Tablet Group: *n* = 54, Placebo Group: *n* = 53. *** MNP Group: *n* = 49, Tablet Group: *n* = 53, Placebo Group: *n* = 53. **** MNP Group: *n* = 47, Tablet Group: *n* = 54, Placebo Group: *n* = 52. AGP = serum alpha-1-acid glycoprotein, CRP = serum C-reactive protein, MNP = multiple micronutrient powder, Zn = zinc, Δ = change from baseline to endline.

## Data Availability

All de-identified study data will be made publicly available at data.mendeley.com upon publication of the manuscript of the primary endpoints of the ZiPT.
